# Supplementary material for: Chemerin-Induced Down-Regulation of Placenta-Derived Exosomal miR-140-3p and miR-574-3p Promotes Umbilical Vein Endothelial Cells Proliferation, Migration, and Tube Formation in Gestational Diabetes Mellitus
Source: Cells. 2022 Nov 1;11(21):3457. doi: 10.3390/cells11213457 (PMC9655594; doi:10.3390/cells11213457)
Supplement: Supplementary file 1 [file cells-11-03457-s001.zip › cells-1860865-supplementary.pdf]

Supplymentary materials

Table S1. Obstetrical characteristics of GDM and normal pregnancy women

Characteristic	GDM (n = 30)	Normal (n = 30)	*p* value
Maternal age (years)	35.10±3.55	33.10±3.82	0.040
Gravidity	2.80±1.42	3.03±1.22	0.498
Parity	0.83±0.53	1.00±0.52	0.226
Pre-pregnancy BMI (kg/m^2)	22.38±2.99	21.54±2.68	0.262
Gestational weight gain (kg)	11.53±3.59	13.83±3.62	0.016
Gestational weeks	38.50±0.68	38.43±0.57	0.682
Birth weight (g)	3410.33±478.30	3357.67±349.25	0.628
fasting plasma glucose (FPG)	4.81±0.54	4.43±0.21	0.001
1-h post-load glucose (1-h PG)	10.60±1.10	7.52±0.96	0.000
2-h post-load glucose (2-h PG)	9.45±0.95	6.63±0.81	0.000

Table S2. Differentially expressed miRNAs with medium and high abundance in placenta-derived exosomes from GDM and normal pregnant women

miRNA ID	Fold Change	*p* Value
hsa-miR-141-5p	0.50	4.00×10^{-3}
hsa-miR-874-3p	0.47	8.78×10^{-3}
hsa-miR-29b-1-5p	0.45	8.90×10^{-3}
hsa-mir-3196	0.30	1.06×10^{-2}
hsa-miR-362-5p	0.62	1.44×10^{-2}

hsa-miR-1246	2.84	1.85×10^{-2}
hsa-miR-518a-3p	0.55	1.90×10^{-2}
hsa-miR-34a-5p	0.52	2.10×10^{-2}
hsa-miR-584-5p	0.54	2.10×10^{-2}
hsa-miR-331-5p	0.65	2.11×10^{-2}
hsa-miR-320a-3p	0.62	2.26×10^{-2}
hsa-miR-22-5p	0.68	2.42×10^{-2}
hsa-miR-30c-1-3p	0.51	2.60×10^{-2}
hsa-miR-519d-3p	0.56	2.73×10^{-2}
hsa-miR-767-5p	0.51	2.78×10^{-2}
hsa-miR-500a-3p	0.72	2.91×10^{-2}
hsa-miR-502-3p	0.72	2.91×10^{-2}
hsa-miR-30b-3p	0.53	3.04×10^{-2}
hsa-miR-766-3p	0.42	3.41×10^{-2}
hsa-miR-519a-2-5p	0.59	3.47×10^{-2}
hsa-miR-27a-5p	0.49	3.52×10^{-2}
hsa-miR-29c-5p	0.56	3.53×10^{-2}
hsa-miR-574-5p	0.66	3.57×10^{-2}
hsa-miR-128-3p	1.56	3.59×10^{-2}
hsa-miR-375-3p	0.55	3.71×10^{-2}
hsa-mir-518b	0.58	3.79×10^{-2}
hsa-miR-548i	0.40	3.80×10^{-2}

hsa-miR-518e-5p	0.64	4.08×10^{-2}
hsa-miR-520f-5p	0.64	4.08×10^{-2}
hsa-miR-140-3p	0.43	4.20×10^{-2}
hsa-miR-362-3p	0.61	4.23×10^{-2}
hsa-miR-195-5p	0.59	4.29×10^{-2}
hsa-miR-1296-5p	0.61	4.45×10^{-2}
hsa-miR-501-3p	0.68	4.57×10^{-2}
hsa-miR-548ai	0.42	4.60×10^{-2}
hsa-miR-561-5p	0.60	4.66×10^{-2}
hsa-miR-574-3p	0.45	4.93×10^{-2}
hsa-miR-526a-3p	0.52	4.99×10^{-2}

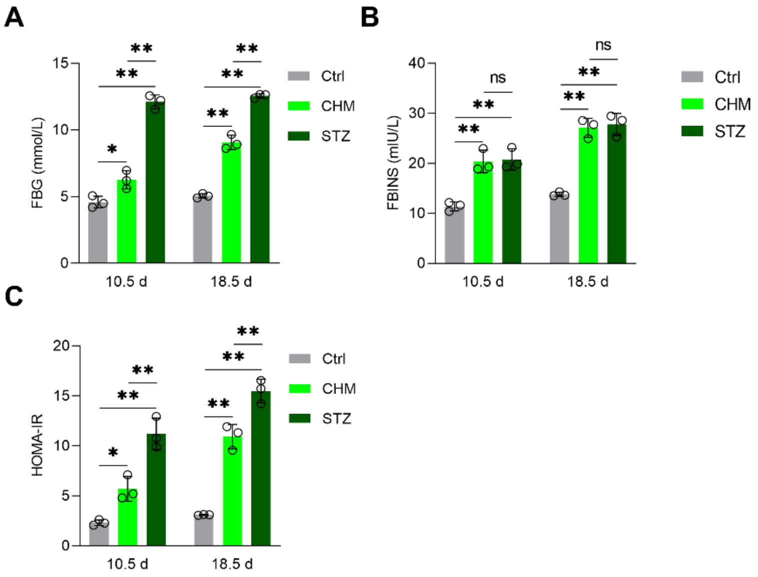

Figure S1. Chemerin-induced diabetic pregnant model. (A) Measurements of fasting blood glucose (FBG) in the day 10.5 and 18.5 after pregnancy of control, chemerin-induced diabetes

mice and STZ-induced diabetes mice. (B) Measurements of fasting blood insulin (FBINS). (C) Homeostasis model of assessment-insulin resistance (HOMA-IR). *$p < 0.05$, ** $p < 0.01$. Ns: no significance.

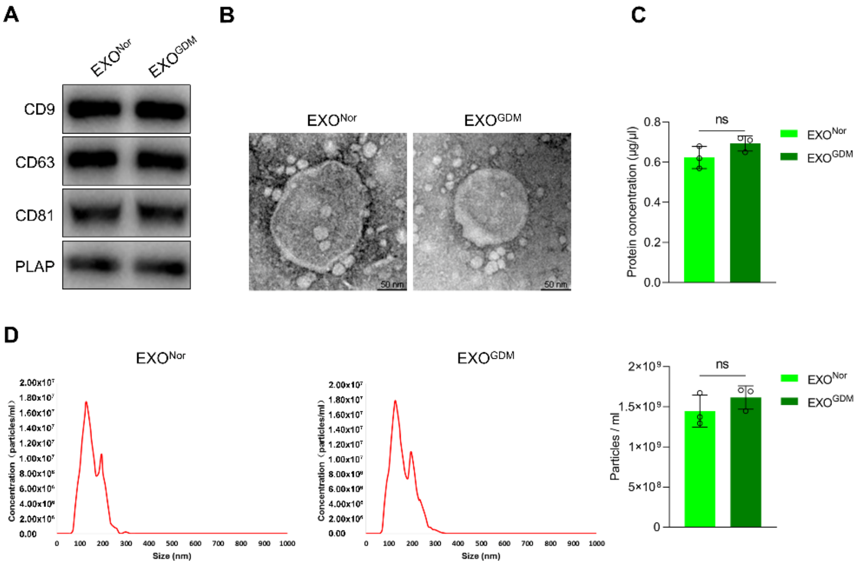

Figure S2. Identification of placenta-derived exosomes from placenta tissue. (A) Protein levels of CD63, CD9 and CD81 and PLAP of placenta-derived exosomes from normal and GDM pregnant women were analyzed by western blotting. (B) Placenta-derived exosomes were observed under electron microscopy. (C) The protein concentration of placenta-derived exosomes from normal and GDM pregnant women. (D) The size distribution and particles of placenta-derived exosomes from normal and GDM pregnant women. Ns: no significance.

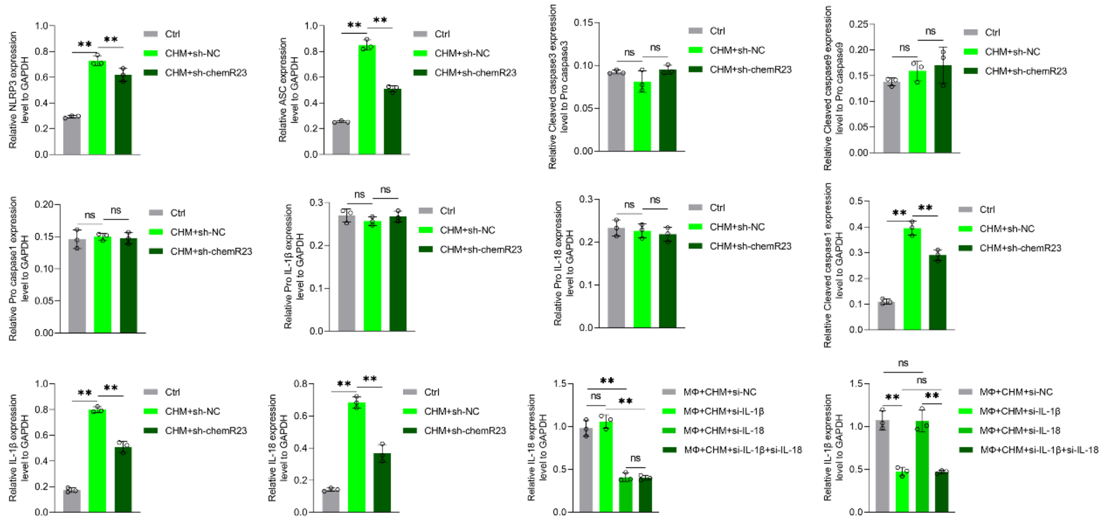

Figure S3. Protein levels of NRLP3, Asc, cleaved caspase 3, cleaved caspase 9, pro caspase 1, pro IL-1β, pro IL-18 in macrophages and levels of caspase 1, IL-1β and IL-18 in the culture supernatants of macrophages. IL-1β and IL-18 were measured of the supernatant from macrophage+CHM+sh-NC, macrophage+CHM+sh-IL-1β, macrophage+CHM+sh-IL-18 and macrophage+CHM+sh-IL-1β+sh-IL-18. ***p* < 0.01. Ns: no significance.

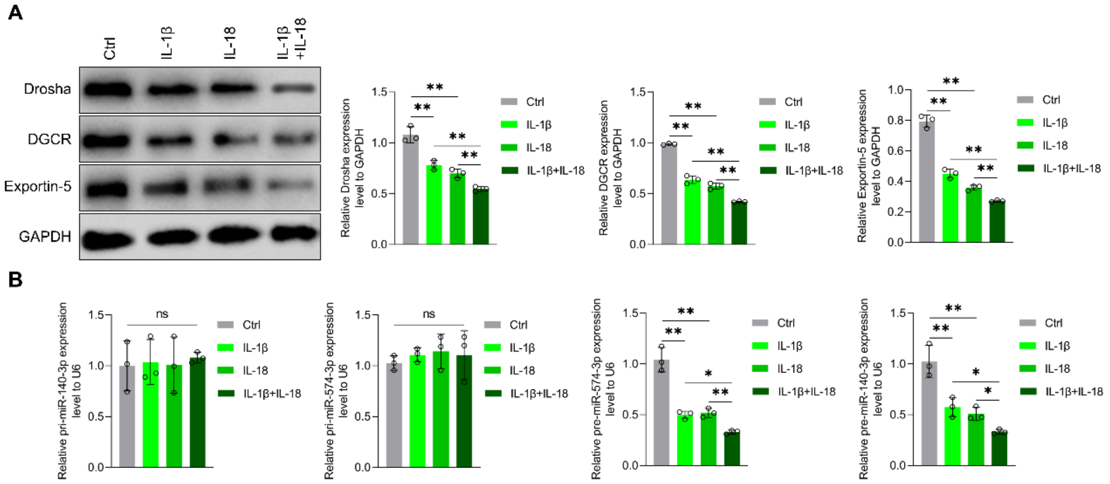

Figure S4. Trophoblast cells were treated with negative control, IL-18, IL-1β or IL-18+IL-1β. (A) Protein levels of Drosha, DiGeorge critical region 8 (DGCR8) and Exportin-5 were measured by western blotting. (B) Relative pri-miR-140-3p, pri-miR-574-3p, pre-miR-140-3p and pre-miR-574-3p expression were measured by qRT-PCR. **p* < 0.05, ** *p* < 0.01. Ns: no significance.

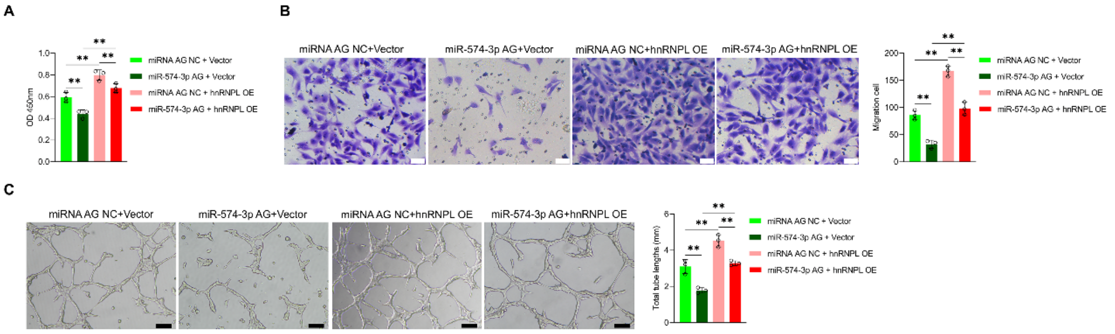

Figure S5. MUVECs were transfected with miRNA AG NC+vector, miR-574-3p AG+vector, miRNA AG NC+hnRNP L overexpression (OE), miR-574-3p AG+hnRNPL OE. (A) The CCK-8 was applied to detect the proliferation of MUVECs. (B) A transwell assay applied to detect the migration of MUVECs (Scale bar: 50 μm). (C) Tube formation assay was applied to detect the tube formation of MUVECs (Scale bar: 100 μm). **$p < 0.01$.
